# Amphotericin B-PEG Conjugates of ZnO Nanoparticles: Enhancement Antifungal Activity with Minimal Toxicity

**DOI:** 10.3390/pharmaceutics14081646

**Published:** 2022-08-07

**Authors:** Saad M. Alshahrani, El-Sayed Khafagy, Yassine Riadi, Ahmed Al Saqr, Munerah M. Alfadhel, Wael A. H. Hegazy

**Affiliations:** 1 Department of Pharmaceutics, College of Pharmacy, Prince Sattam Bin Abdulaziz University, Al-Kharj 11942, Saudi Arabia; 2 Department of Pharmaceutics and Industrial Pharmacy, Faculty of Pharmacy, Suez Canal University, Ismailia 41522, Egypt; 3 Department of Pharmaceutical Chemistry, College of Pharmacy, Prince Sattam Bin Abdulaziz University, Al-Kharj 11942, Saudi Arabia; 4 Department of Microbiology and Immunology, Faculty of Pharmacy, Zagazig University, Zagazig 44519, Egypt; 5 Department of Pharmaceutical Sciences, Pharmacy Program, Oman College of Health Sciences, Muscat 113, Oman

**Keywords:** amphotericin, PEGylation, ZnO, metallic nanoparticles, antifungal

## Abstract

Amphotericin B (AMB) is commonly used to treat life-threatening systemic fungal infections. AMB formulations that are more efficient and less nephrotoxic are currently unmet needs. In the current study, new ZnO-PEGylated AMB (ZnO-AMB-PEG) nanoparticles (NPs) were synthesized and their antifungal effects on the *Candida* spp. were investigated. The size and zeta potential values of AMB-PEG and ZnO-AMB-PEG NPs were 216.2 ± 26.9 to 662.3 ± 24.7 nm and −11.8 ± 2.02 to −14.2 ± 0.94 mV, respectively. The FTIR, XRD, and EDX spectra indicated that the PEG-enclosed AMB was capped by ZnO, and SEM images revealed the ZnO distribution on the surface NPs. In comparison to ZnO-AMB NPs and free AMB against *C.albicans* and *C.neoformans*, ZnO-AMB-PEG NPs significantly reduced the MIC and MFC. After a week of single and multiple dosage, the toxicity was investigated utilizing in vitro blood hemolysis, in vivo nephrotoxicity, and hepatic functions. ZnO-AMB-PEG significantly lowered WBC count and hematocrit concentrations when compared to AMB and ZnO-AMB. RBC count and hemoglobulin content, on the other hand, were unaltered. ZnO-AMB-PEG considerably lowered creatinine and blood urea nitrogen (BUN) levels when compared to AMB and ZnO-AMB. The difference in liver function indicators was determined to be minor by all formulae. These findings imply that ZnO-AMB-PEG could be utilized in the clinic with little nephrotoxicity, although more research is needed to determine the formulation’s in vivo efficacy.

## 1. Introduction

Polyene antifungal amphotericin B was first isolated from *Streptomyces nodosus*. Amphotericin B (AMB) binds to the fungal cell membrane’s ergosterol, holing it and allowing cell components to leak out, which results in cell death [1,2]. AMB confers the most reliable and broad-spectrum antifungal effects, especially in the rising prevalence and changing spectrum of invasive fungal infections [2]. The spectrum of AMB is extended to be used as antiprotozoal in life-threatening infections such as primary amoebic meningoencephalitis [3], and visceral leishmaniasis [4]. Despite the clinical importance of AMB as the antifungal drug of choice for most systemic infections, the development of nephrotoxicity is a limiting factor for its use [1,2,5]. In this direction, several approaches have been proposed to formulate AMB in low concentrations to avoid nephrotoxicity [2].

Because AMB is amphiphilic, it is poorly soluble in most organic solvents and almost insoluble in water under physiological conditions of pH = 6.7. AMB is typically given intravenously (IV) as a complex with sodium deoxycholate, which acts as a dispersant, forming water-soluble micellar colloidal complexes with AMB [6]. While numerous formulations have been shown to be effective in the clinic, their drawbacks have fueled interest in generating better formulations and semisynthetic AMB derivatives [7,8]. Various AMB conjugates to biomolecules and water-soluble polymers have been reported for this purpose [8].

Being biologically inert, non-immunogenic and nontoxic, PEG has been frequently employed as a modifier for both protein and non-protein small drug molecules. Covalent attachment of this extremely hydrophilic polymer improves the water solubility of many drugs and can bestow other desired features such as decreased immunogenicity, enhanced pharmacokinetics, and targeted delivery [9]. Many reports have emphasized the potential role of PEG for enhancing the aqueous solubility and decreasing the toxicity of AMB when conjugated to it [10,11,12,13]. Sedlak, et al. used a carbamate linker to attach PEG to AMB via the amine function of the mycosamine [10,11]. This AMB−PEG conjugate is thought to act as a prodrug, releasing free AMB in vivo upon carbamate function hydrolysis [11]. Greenwald described a similar type of compound in which the PEG and AMB moiety were combined. Through a cascade reaction, enzymatic hydrolysis of the AMB−PEG results in the rapid release of free AMB [12,13].

By expanding the surface area, promoting drug release, lowering the dose required, and improving drug solubility and bioavailability, nanoparticles (NPs) have exhibited antibacterial efficacy against a variety of multiple drug resistant (MDR) bacteria [14,15,16,17]. Dendrimers, liposomes, metallic nanoparticles, and polymeric nanoparticles have all been investigated as delivery vehicles. Metal nanoparticles have inherent physicochemical and optical features that make them suitable for a wide range of applications [18,19]. Zinc oxide NPs (ZnO-NPs) have been used on a nano- and microscale in various formulations and have been found to be safe for human cells [20]. ZnO-NPs have unusual semiconducting characteristics, which account for their widespread use in electronics and biomedicine. ZnO-NPs have natural antibacterial properties and have been approved by the FDA as biocompatible antimicrobials [18]. Furthermore, ZnO-NPs are inexpensive, and their ease of functionalization makes the development of antibacterial formulations feasible [21].

ZnO-AMB-PEG NPs was synthesized in this study, and following successful synthesis, the NPs and their conjugates were characterized using a variety of techniques including zeta sizer and zeta potential analysis, FTIR, XRD, SEM, and EDX. The antifungal properties of the NPs and drug-NP conjugates were also assessed. Finally, hematological, and biochemical tests were used to determine their cytotoxicity/viability.

## 2. Materials and Methods

### 2.1. Materials

The ZnO nanoparticle was purchased from Sigma-Aldrich (Taufkirchen, Germany). Amphotericin B (AMB) was purchased from Beijing Mesochem Technology Co., Ltd. (Beijing, China). Polyethylene glycol (PEG, 40 KDa) was purchased from Sigma-Aldrich (St. Louis, MO, USA). Tween-80 was purchased from Merck Schuchardt OHG (Hohenbrunn, Germany). The used media RPMI 1640 and morpholinepropanesulfonic acid (MOPS) were purchased from Sigma-Aldrich (St. Louis, MO, USA). Sabouraud dextrose agar (SDA) was purchased from Oxoid (Hampshire, UK). Levels of the liver enzymes and kidney functions, aspartate transaminase (AST), alanine transaminase (ALT), and creatinine assay kits were purchased from Sigma-Aldrich (St. Louis, MO, USA). Blood urea nitrogen (BUN) colorimetric detection kit was ordered from Thermo Fisher Scientific (Waltham, MA, USA). All other chemicals and solvent were purchased from Sigma-Aldrich.

### 2.2. Preparation of AMB-PEG

Conjugation of PEG with AMB was carried out according to the literature [13]. A mixture of AMB (210 mg, 0.22 mmol, 3% mass ratio) with PEG (8 g, 0.2 mmol, 97% mass ratio) was reacted in dry DMF (5 mL) in the presence of pyridine (5 mL) for 24 h under inert atmosphere and with exclusion of light. After completion, the mixture was poured into cold ether. The crude obtained was filtered and dissolved in methanol (5 mL) and precipitated by ether (100 mL). The final product was collected by filtration and dried in a vacuum desiccator.

### 2.3. Preparation of ZnO-AMB-PEG Nanoparticles

The formulated nanoparticles were prepared by the nanoemulsification method [22]. The ZnO nanoparticles (2 g, 83% mass ratio) were suspended in water at concentration (2%) and sonicated for 30 min. Then, AMB-PEG (0.4 g, 17% mass ratio) was dissolved in distilled water (50 mL) containing ZnO nanoparticles suspension and were mixed by magnetic stirrer for 1 h. Then, Tween 80 (0.2 mL) as an emulsifier and Cetrimonium bromide (0.8 g) as reducing agent were added and stirred for 15 min. The mixture was homogenized by ultrasound (Ultrasonic Procesor model UP200Ht) for 15 min. Then the mixture was dried at 25 °C for 72 h.

### 2.4. Measurement of Particle Size, Zeta Potential (ZP), and Polydispersity Index (PDI)

The mean particle size, ZP, and PDI of the AMB-PEG, and ZnO-AMB-PEG were measured using a Malvern particle size analyzer (Zetasizer Nano ZS; Holtsville, NY, USA) maintained at 25 ± 1 °C. The light scattering angle was set at 90 °C. The SLNs were dispersed in distilled water at a ratio of 1:200, which resulted in an aqueous dispersion. The AMB-PEG, and ZnO-AMB-PEG dispersions were sonicated for 5 min, then transferred to a disposable transparent plastic cuvette for particle size and PDI measurements. The same procedure was followed for measurement of ZP, except the AMB-PEG, and ZnO-AMB-PEG dispersions were transferred to a glass electrode sample holder.

### 2.5. Fourier-Transform Infrared (FTIR) Spectroscopy

FTIR spectra of AMB, ZnO, PEG, AMB-PEG, and ZnO-AMB-PEG were recorded from 400 to 4000 cm^−1^ using the KBr pellet technique (4100 Jasco FTIR spectrophotometer, Tokyo, Japan). The spectra were interpreted by evaluating the different vibrational and functional peaks.

### 2.6. X-ray Diffraction (XRD)

X-ray diffraction analysis was employed to detect the characterization of pure ZnO, pure AMB-PEG and AMB-PEG doped ZnO. The X-ray diffraction patterns of the ZnO nanoparticles, as-prepared ZnO-AMB-PEG nanocomposite and AMB-PEG were performed using an XRD Ultima IV X-ray diffractometer equipped with a conventional X-ray source Cu Kα radiation (λ: 1.54056 Å).

### 2.7. Morphological Analysis by Scanning Electron Microscope (SEM)

The surface morphology of the ZnO-AMB-PEG particles was inspected by SEM (Inspect F50 SEM, Netherlands). The ZnO-AMB-PEG (1–2 mg) was suspended in 1 mL of purified water. A 50 μL of suspension was smeared on the aluminum stubs, using a two-sided carbon adhesive tape and sputter-coated with conductive gold palladium. A circular coverslip was gently placed over the stub to enable an even distribution of the sample suspension. A uniform conductivity was maintained by using a silver paint lining that was applied to the edges of the coverslip to fill its narrow spacing with the stub. The images were viewed with an EVO LS 10 (Carl Zeiss, Brighton, Germany) SEM, operating at an accelerating voltage of 30.0 kV under high vacuum.

### 2.8. EDX Elemental Analysis

The EDX system mounted on the Quanta 200 SEM was used for element identification and performing the quantitative analysis. The elemental analysis was performed on the surface of the ZnO-AMB-PEG. No secondary treatment or processing was necessary.

### 2.9. In Vitro Antifungal Assay

The broth microdilution method was used to assess the antifungal susceptibility of free AMB, and ZnO, ZnO-AMB, and ZnO-AMB-PEG nanoparticles against *Candida albicans* ATCC 64548 and *Cryptococcus neoformans* ATCC 90112 according to the Clinical Laboratory and Standards Institute Guidelines (CLSI, 2015) [23,24,25,26]. The nanoparticle solutions were serially diluted in sterile saline to obtain dilutions ranging from 5.0 to 0.00625 µg/mL. Aliquots (100 µL) from different dilutions of different preparations were transferred to the wells of 96-well microtiter plate. Fresh cultures of the tested strains were suspended in RPMI 1640 provided with L-glutamine and MOPS at pH 7.0 to prepare suspensions with a turbidity approximating that of 0.5 McFarland Standard. The fungal suspensions were diluted 1:100 with RPMI 1640 to obtain cell density of 1 × 10^5^ CFU/mL. Of the prepared fungal suspensions, 100 µL was added to all dilutions of nanoparticle preparations. The microtiter plates were incubated at 37 °C for 24 h for *C. albicans*, and 48 h for *C. neoformans.* The minimum inhibitory concentration (MIC) was considered as the lowest concentration that inhibited the visible turbidity. The experiment was repeated six times and both positive control (inoculated fungi in broth without any addition) and negative control (sterile broth without fungi) were included in the experiment. The minimum fungicidal concentration (MFC) was determined according to [23]. Suspensions from the wells (100 µL), which did not show any growth after incubation during MIC assays were spread on SDA plates and incubated at 37 °C for 48 h for *C. albicans*, and 72 h for *C. neoformans.* The MFC was considered as the lowest concentration which completely inhibited the fungal growth.

### 2.10. In Vivo Studies

#### 2.10.1. Animals

Male Wistar rats weighing 180–220 g were used in this study. The animals were housed in rooms with controlled temperature (23 ± 1 °C) and relative humidity (55 ± 5%) and were allowed free access to water and food during acclimatization. Animals were fasted for 24 h before the experiments but could drink water ad libitum. The animal study protocol was approved by the Ethical Committee, Pharmacy College, Prince Sattam Bin Abdulaziz University, Al-Kharj, KSA (approval number: PHARM-11-10-2021).

#### 2.10.2. Hematological and Biochemical Evaluations of AMB Preparations

Blood parameters including count of leucocytes (WBC) and red blood cell (RBC), and hemoglobin concentration (Hb), and hemocrit percentage were employed to evaluate the hematological toxicity associated with AMB. Furthermore, the kidney functions were assessed by measuring blood urea nitrogen (BUN) and serum creatinine. Hepatic functions were evaluated by the measure of aspartate transaminase (AST) and alanine transaminase (ALT) [27,28,29]. Four groups of male Wistar rats, each composed of 5 rats weighing approximately 200 g were used in this study. Three groups were intraperitoneally injected with free AMB, ZnO-AMB or ZnO-AMB-PEG (10 mg/kg body weight) for a continuous seven days. The fourth group was left uninjected as control group. On the eighth day, all rats were anesthetized and euthanized by decapitation for hematological and biochemical assays [30,31].

## 3. Results and Discussion

### 3.1. Preparation of AMB-PEG

The elucidation of the total proton and carbon numbering and environment was carried out by recording the ^13^C-NMR spectra using NMR BRUCKER-PLUS instrument. ^13^C-NMR (C_5_D_5_N; 67.8 MHz) δ 156.82; 154.01; 151.32; 136.58; 136.13; 135.01; 134.24; 134.05; 133.91; 133.77; 133.15; 132.52; 132.42; 132.11; 129.35; 128.02; 120.92; 97.96; 97.54; 77.31; 76.21; 75.62; 74.76; 74.23; 72.98–68.94 (Polyethylene Glycol); 69.07; 67.04; 66.58; 66.32; 65.47; 62.51; 61.23; 58.85; 58.01; 48.23; 44.98; 44.76; 43.39; 42.65; 40.21; 40.01; 37.81; 36.12; 31.48; 18.23; 18.01; 16.77; 13.01.

### 3.2. Characterization of AMB Nanoparticles

Particle size, PDI, and ZP data for the AMB nanoparticles are summarized in Table 1. The AMB nanoparticles (AMB-PEG, and ZnO-AMB-PEG) ranged in size from 216.2 ± 26.9 (AMB-PEG) to 662.3 ± 24.7 nm (ZnO-AMB-PEG), which was within the nanoparticulate range of ≤1000 nm. The size of the nanocomposites increased with the addition of ZnO. The PDI values of the NPs ranged from 0.315 to 0.525. Values less than 1.0 indicated that the particles were suitable for analysis using differential light scanning analysis. Larger PDI values can occur due to the capping of ZnO with AMB-PEG.

The ZP values were −11.8 ± 2.02, and −14.2 ± 0.94 mV for AMB-PEG, and ZnO-AMB-PEG, respectively (Table 1). These results demonstrated that the nanocomposites produced in this study were a stable system under dynamic conditions. Negative ZP values may have been due to slightly negatively charged PEG and ZnO on the particle surfaces at neutral pH. Negative ZP values of ZnO-AMB-PEG almost similar to those of AMB-PEG indicated that the charge of ZnO was insignificant in the interactions with AMB within the nanocomposites.

The antifungal activity of AMB in the ZnO-AMB-PEG nanocarrier formulation in this study was governed by several factors including the physicochemical properties of the carrier such as size, surface area, charge, and the drug solubility, and depended on the chemical composition of the ZnO and PEG conjugated carrier. Among these factors, the influence of the size and the charge have been widely studied. Nanoparticles of approximately 300–600 nm in size exhibit potential antifungal activity, where they have a significantly sustained release of drug. Moreover, the drug absorption and biological activity depend also on the morphology of nanoparticles, biological adhesiveness, and degradation in vivo. In addition, the biodegradable PEG polymeric and inorganic porous ZnO carriers represent the alternative delivery system that enhance the absorption of AMB for antifungal application [32].

### 3.3. FTIR Spectral Analysis

**Figure 1:**
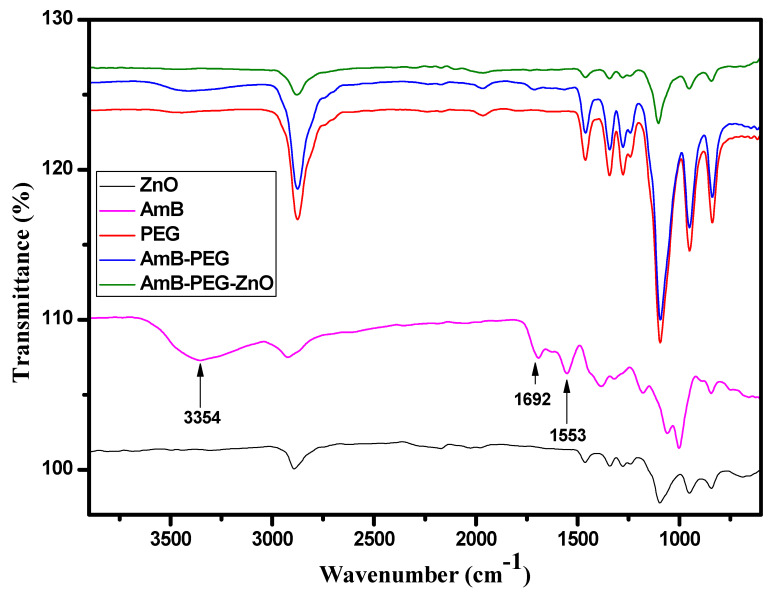
FTIR spectra of ZnO, AMB, PEG, AMB-PEG, and ZnO-AMB-PEG composites.

To explore the changes occurring to AMB after being sequentially mixed with PEG and ZnO, the FTIR spectra of ZnO, AMB, AMB-PEG as well as ZnO-AMB-PEG are shown in Figure 1. The spectrum of pure AMB exhibited the typical reported bands for this molecule located at 3392, 2935, 1692 and 1559 cm^−1^ and assigned to the O-H, C-H, C=O and C=C stretching vibrations, respectively [33]. The AMB-PEG composite preserved the characteristic peaks of the pure PEG. However, after conjugation with PEG polymer chains, the three bands located at 3392 cm^−1^, 1692 cm^−1^ and 1559 cm^−1^ corresponding to the O-H, C=O and C=C stretching vibrations of AMB were either broadened or vanished. These observations indicated the formation of a single compound from AMB and PEG polymer because of PEG polymer encapsulation. On the other hand, after mixing with ZnO, all the specific bands associated with AMB disappeared and the FTIR spectrum was fairly similar to that of pure ZnO, implying that the PEG-encapsulated AMB was further capped by ZnO nanoparticles as confirmed by the SEM analysis.

### 3.4. X-ray Diffraction (XRD) Characterization of Pure ZnO, Pure AMB-PEG and AMB-PEG Doped ZnO

**Figure 2:**
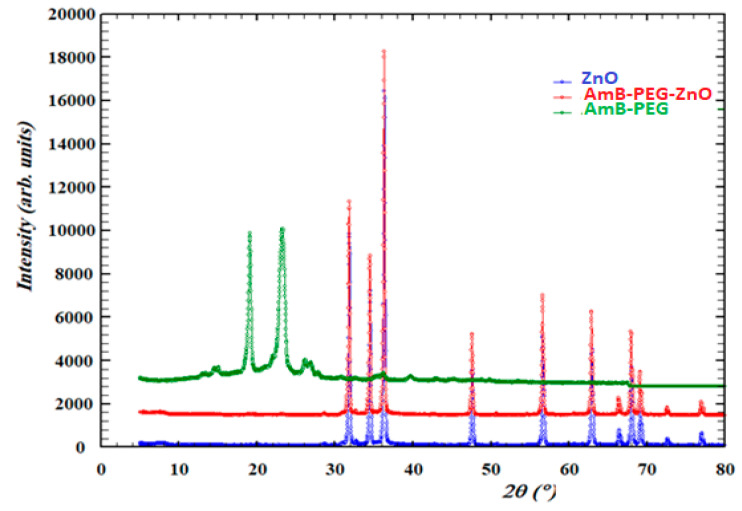
XRD patterns of pure ZnO, AMB-PEG and AMB-PEG-ZnO nanoparticles.

Figure 2 shows that the pure ZnO nanoparticles, ZnO-AMB-PEG nanocomposite and AMB-PEG were characterized using powder X-ray diffraction (PXRD). The figure below shows the comparison between XRD patterns of these compounds. The AMB-PEG loaded ZnO and pure ZnO nanoparticles presented the same phase and AMB-PEG phase did not appear in the spectra of the prepared nanocomposite, therefore it could be explained that ZnO covered the AMB-PEG.

### 3.5. Morphological Analysis: SEM and EDX

**Figure 3:**
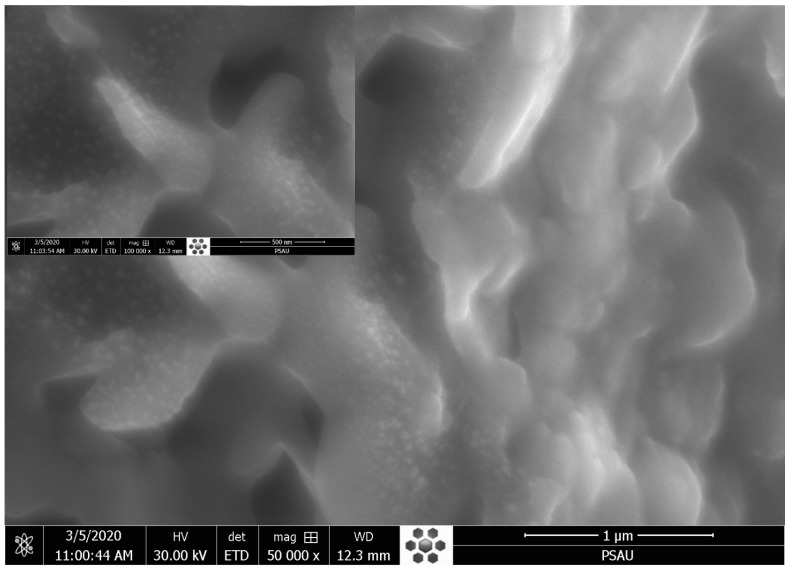
SEM image of AmB-PEG-ZnO sample.

**Figure 4:**
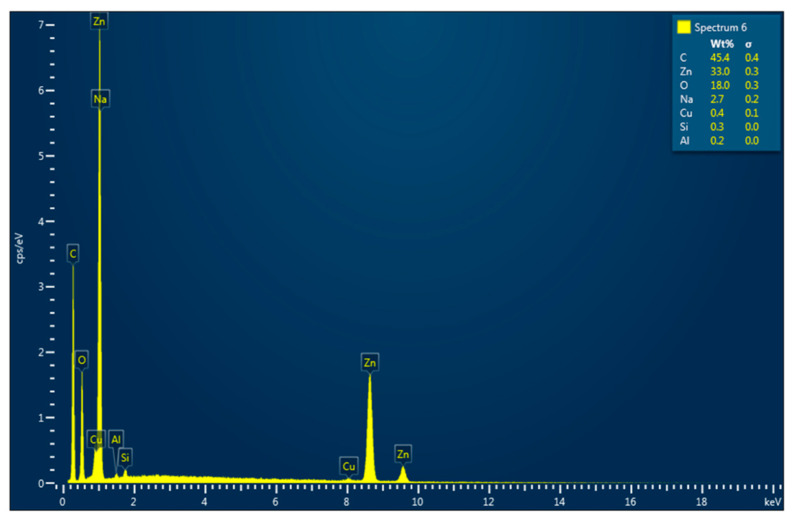
EDX spectrum of ZnO-AMB-PEG sample.

The SEM micrographs of the ZnO-AMB-PEG composite are illustrated in Figure 3. The SEM image showed well-distributed ZnO nanoparticles on the surface of the sample. Moreover, the EDX spectrum (Figure 4) revealed that the main constituents of the sample were zinc, oxygen, and carbon. The high intensity of the zinc and oxygen peaks supported the ZnO capping of the AMB-PEG composite.

### 3.6. Antifungal Susceptibility of Nanoparticles

**Figure 5:**
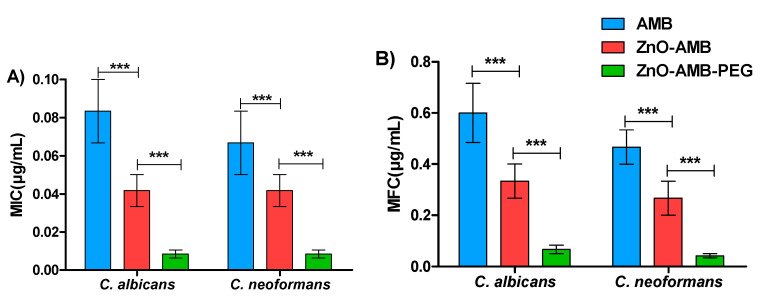
Antifungal susceptibility of ZnO-AMB-PEG compared to ZnO-AMB and free AMB. (**A**) Minimum inhibitory concentration (MIC), and (**B**) minimum fungicidal concentration (MFC) of tested preparations against *C. albicans* and *C. neoformans*. Mann–Whitney U analysis demonstrates a statistically significant difference (*** *p* < 0.001) between *C. albicans* and *C. neoformans* in their susceptibilities (MICs and MFCs) to ZnO-AMB-PEG, ZnO-AMB and free AMB.

The antifungal activities of free AMB, and ZnO, ZnO-AMB, and ZnO-AMB-PEG nanoparticles against *C. albicans* and *C. neoformans* were evaluated and expressed as MIC and MFC (Table 2). To demonstrate the statistical significance, Mann–Whitney U analysis was used. The ZnO-AMB-PEG nanoparticles significantly decreased the MIC and MFC in comparison to ZnO-AMB nanoparticles and free AMB (Figure 5).

To evaluate the effectiveness of different AMB preparations, the MICs and MFCs to *C. albicans* and *C. neoformans* were determined against ZnO-AMB-PEG, ZnO-AMB, ZnO nanoparticles, and free AMB as an indication to antifungal effectiveness. The PEGylated ZnO-AMB inhibited or killed fungal cells at significantly low concentrations in comparison with free AMB or ZnO-AMB. These in vitro findings were in compliance with previous studies that showed the efficient diffusion of PEGylated nanoparticles in liquid media to target fungal cells [23,34,35,36]. The superior antifungal activity of ZnO-AMB-PEG, compared to other formulations, might be attributed, at least in part, to the enhanced colloidal stability of ZnO-AMB-PEG imparted by the presence of the hydrophilic polymer, PEG, which prevents the aggregation of the formulated nanoparticles, and consequently, eases the penetration of ZnO-AMB-PEG nanoparticles through the pores of fungal cell membranes. In addition, the presence of PEG has been repeatedly reported to prevent the adsorption of the components of biological fluids (e.g., proteins, electrolytes, lipids, and metabolites) leading to the formation of a protein corona, which may affect their antimicrobial activity [37,38].

### 3.7. In Vivo Toxicity Assay

**Figure 6:**
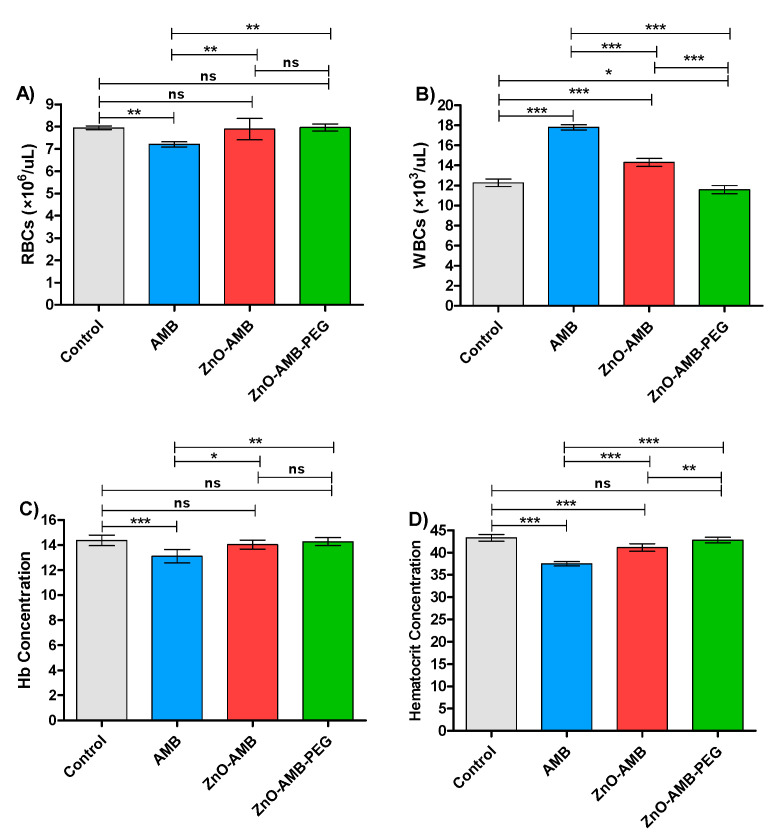
Hematological parameters. Four groups of rats (*n* = 5) were injected intraperitonially with 10 mg/kg seven doses of free AMB, ZnO-AMB or ZnO-AMB-PEG or kept uninjected, then hematological parameters were evaluated (A; RBCs concentration, B; WBCs concentration, C; Hb concentration, D; Hematocrit concentration). The assays were conducted in triplicate and one-way ANOVA test followed by Tukey’s multiple comparison post hoc test were used to attest the significance. The PEGylated ZnO-AMB did not show any significant difference in hematological parameters in comparison to control group levels, except ZnO-AMB-PEG significantly increased the WBCs in comparison to control group. Furthermore, ZnO-AMB-PEG significantly decreased the WBCs count and hematocrit concentrations in comparison to ZnO-AMB. * = *p* < 0.05, ** = *p* < 0.01 and *** = *p* < 0.001, ns = non-significant.

Four groups of rats were intraperitoneally injected with 10 mg/kg of free AMB, ZnO-AMB or ZnO-AMB-PEG or kept not-injected for seven successive days. The blood was withdrawn for hematological examinations (Table 3). The tests were conducted in triplicates and one-way ANOVA test followed by Tukey’s multiple comparison post hoc test were used to evaluate the significance where *p* < 0.05 was considered significant (Figure 6). The PEGylated ZnO-AMB significantly decreased the WBC count and hematocrit concentrations to the measures of control groups in comparison to ZnO-AMB. Moreover, the RBC count and hemoglobulin concentration were not influenced significantly in ZnO-AMB and ZnO-AMB-PEG groups in comparison to the control group. On the other hand, the hematological parameters were significantly influenced in the free AMB group in comparison to the control group.

**Figure 7:**
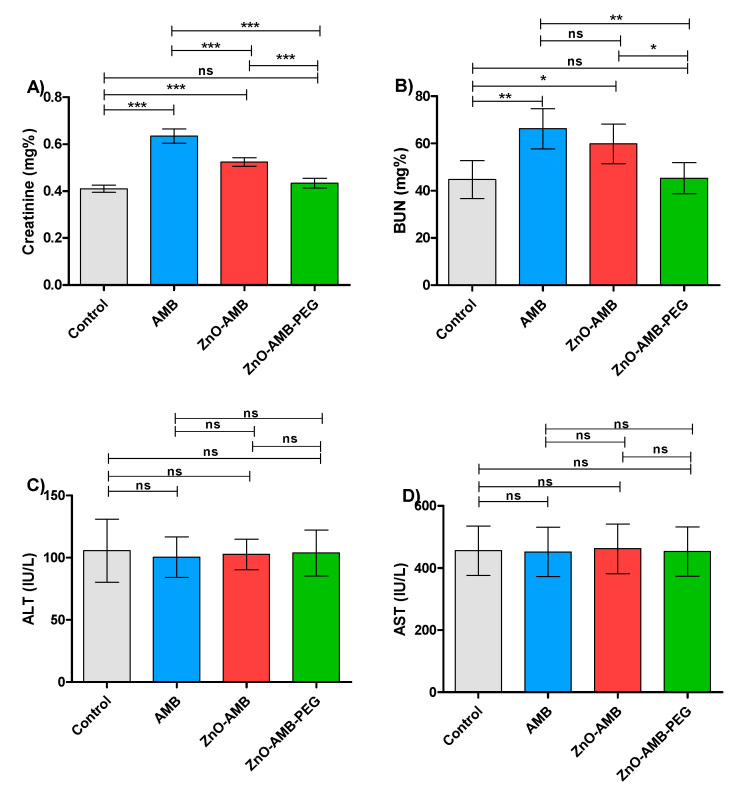
Biochemical parameters. Four groups of rats (*n* = 5) were injected intraperitonially with 10 mg/kg seven doses of free AMB, ZnO-AMB or ZnO-AMB-PEG or kept uninjected, then, kidney and liver functions were evaluated (**A**; Creatinine concentration, **B**; BUN concentration, **C**; ALT concentration, **D**; AST concentration). The assays were conducted in triplicate and one-way ANOVA test followed by Tukey’s multiple comparison post hoc test were used to attest the significance. The PEGylated ZnO-AMB did not significantly increase the levels of kidney function markers creatinine and BUN in comparison to control group. Meanwhile, ZnO-AMB-PEG significantly decreased the levels of kidney markers in comparison to ZnO-AMB and free AMB. One the other hand, there was insignificance between liver enzymes ALT and AST in different groups. * = *p* < 0.05, ** = *p* < 0.01 and *** = *p* < 0.001, ns = non-significant.

AMB is a well-known antifungal for its nephrotoxicity side effects; in this direction it was aimed to evaluate the effect of different preparations on the kidney functions (Table 4). The PEGylated ZnO-AMB reduced the levels of kidney function markers creatinine and BUN almost to control group levels and significantly lower than ZnO-AMB or free AMB (Figure 7A,B). These findings indicate that the ZnO-AMB-PEG significantly lowered the burden on the kidney to more safe levels. Furthermore, the influence of the tested preparations was evaluated on the liver by assessing the liver enzymes in different animal groups. There was no significant difference between ALT and AST levels in the different groups (Figure 7C,D). The experiments were performed in triplicate and one-way ANOVA test followed by Tukey’s multiple comparison post hoc test were used to evaluate the significance where *p* < 0.05 was considered significant.

The toxicity of ZnO-AMB-PEG, ZnO-AMB nanoparticles, and free AMB was tested in animals. In contrast to ZnO-AMB and free AMB, which changed the hematological parameters over control rats, PEGylated ZnO-AMB retained the hematological parameters of the uninjected control rats. Previous research has shown that successfully integrating nanoparticles into biomedical applications necessitates modulating their surface properties so that the interaction with biological systems is regulated to minimize toxicity for biological function [39]. The interface between blood cells and nanoparticles is a fast and dynamic bio-organic process that ultimately redesigns the properties of the blood cell, which has a direct impact on biological activity [40,41]. The resulting nanoparticles exhibited surface corona as well as free radical-scavenging and enzyme activities with limited cytotoxicity and genotoxicity. In the current study, we conjugated the AMB-PEG moiety on the surface of ZnO nanoparticles and then measured RBCs, WBCs, hemoglobin, and hematocrit concentration in rats. The findings show that developed ZnO nanoparticles conjugated to AMB-PEG have a potential designed nanoparticle surface for biological applications.

Furthermore, the measurement of creatinine and BUN levels were employed to assess the kidney functions of the injected rats with different preparations. The present findings showed that the creatinine and BUN levels were significantly lowered in the case of injection with ZnO-AMB-PEG in comparison to free AMB or ZnO-AMB. This indicates the significant ability of ZnO-AMB-PEG to reduce the amphotericin-induced nephrotoxicity. On the other hand, there was no significant difference between levels of liver function markers in control groups and all other groups. These results emphasize the possibility of clinical application of PEGylated ZnO-AMB at lower concentrations with minimal nephrotoxicity.

Amphotericin B is the drug of choice for the treatment of systemic fungal infections and its mechanism is based on interaction with ergosterol of the fungal cell membrane leading to cell death [42]. Due to Amphotericin B affinity to sterols, it binds to cholesterol on the mammalian cell membranes leading to several side effects, mainly nephrotoxicity [43]. Thus, its clinical efficacy is restricted by the toxicity occurring after a short time of treatment, and hence the development of an effective and less toxic Amphotericin B formulation would be essential. The hydrophilic polymer, polyethylene glycol (PEG), has been widely employed to improve the aqueous solubility of many therapeutic agents including AMB [10,13]. In addition, PEG has been reported to bestow conjugated molecules with other desired features such as decreased immunogenicity and enhanced pharmacokinetic profiles [9]. Furthermore, it is widely recognized that nanoparticles, by virtue of their small size, could guarantee efficient penetration and accumulation into the targeted body tissues, decreasing drug effects and requiring less frequent administration [44,45]. Accordingly, the loading of AMB-PEG conjugates onto ZnO nanoparticles may therefore be a viable strategy to further improve both the solubility and therapeutic index of AMB, as manifested by a remarkable decrease in MIC and MFC in ZnO-AMB-PEG.

## 4. Conclusions

Amphotericin B (AMB) is commonly used to treat systemic fungal infections. However, AMB has many drawbacks, associated primarily with its high toxicity and its poor water solubility that limit its usefulness. Here, we report the preparation and evaluation of ZnO-AMB-PEG nanoparticles. ZnO-AMB-PEG nanoparticles showed potent activity against in vitro antifungal activity against *C. albicans* and *C. neoformans* and significantly decreased MIC and MFC in comparison to ZnO-AMB nanoparticles and free AMB. The toxicity of the ZnO-AMB-PEG, ZnO-AMB nanoparticles, and free AMB were in vivo evaluated. The PEGylated ZnO-AMB retained the hematological parameters as those in uninjected control rats, in contrast to ZnO-AMB and free AMB which changed the hematological parameters over control rats. Moreover, the creatinine and BUN levels were significantly lowered in the case of injection with ZnO-AMB-PEG in comparison to free AMB or ZnO-AMB. This indicates the significant ability of ZnO-AMB-PEG to reduce the amphotericin-induced nephrotoxicity. On the other hand, there was insignificant difference between the levels of liver function markers in control groups and all other groups. These results emphasize the possibility of clinical applications for PEGylated ZnO-AMB at lower concentrations with minimal nephrotoxicity; however, further study is needed to investigate the in vivo efficacy of the developed formulation.

## Figures and Tables

**Table 1:** Particle characterization of AMB nanoparticles.

Formulations	Size (nm ± SD)	*PDI*	*ZP* (mV ± SD)
AMB-PEG	216.2 ± 26.9	0.315	−11.8 ± 2.02
ZnO-AMB-PEG	662.3 ± 24.7	0.525	−14.2 ± 0.94

**Table 2:** Antifungal activity of AMB nanoparticles.

Preparation	*C. albicans*		*C. neoformans*	
	MIC (µg/mL)	MFC (µg/mL)	MIC (µg/mL)	MFC (µg/mL)
**ZnO**	>5	>5	>5	>5
**AMB**	0.1	0.6	0.05	0.4
**ZnO-AMB**	0.05	0.4	0.05	0.2
**ZnO-AMB-PEG**	0.00625	0.05	0.00625	0.05

**Table 3:** Hematological parameters on post-injection effects.

Preparation	RBCs (×10^6^/μL)	WBCs (×10^3^/μL)	Hb (g%)	Hematocrit (%)
**Control**	7.99 ± 0.15	12.45 ± 1.65	14.15 ± 0.55	43.45 ± 0.99
**AMB**	7.09 ± 0.61	17.90 ± 2.46	13.22 ± 0.95	37.01 ± 2.68
**ZnO-AMB**	7.49 ± 1.21	14.26 ± 1.18	13.99 ± 0.50	40.92 ± 2.35
**ZnO-AMB-PEG**	7.96 ± 0.57	11.56 ± 2.08	14.50 ± 0.40	42.80 ± 1.25

**Table 4:** Biochemical parameters on post-injection effects.

Preparation	Kidney Functions		Liver Functions	
	Creatinine (mg%)	BUN (mg%)	ALT (IU/L)	AST (IU/L)
**Control**	0.40 ± 0.10	44.88 ± 10.65	107.90 ± 30.55	460.89 ± 111.99
**AMB**	0.63 ± 0.11	66.52 ± 12.46	100.49 ± 20.95	453.15 ± 120.19
**ZnO-AMB**	0.53 ± 0.21	53.26 ± 11.18	102.08 ± 19.50	470.26 ± 131.13
**ZnO-AMB-PEG**	0.43 ± 0.12	47.86 ± 13.08	104.28 ± 25.40	455.49 ± 100.99

## Data Availability

All data can be found within the article.
